# Integrated molecular and pharmacological characterization of patient-derived xenografts from bladder and ureteral cancers identifies new potential therapies

**DOI:** 10.3389/fonc.2022.930731

**Published:** 2022-08-11

**Authors:** Hervé Lang, Claire Béraud, Luc Cabel, Jacqueline Fontugne, Myriam Lassalle, Clémentine Krucker, Florent Dufour, Clarice S. Groeneveld, Victoria Dixon, Xiangyu Meng, Aurélie Kamoun, Elodie Chapeaublanc, Aurélien De Reynies, Xavier Gamé, Pascal Rischmann, Ivan Bieche, Julien Masliah-Planchon, Romane Beaurepere, Yves Allory, Véronique Lindner, Yolande Misseri, François Radvanyi, Philippe Lluel, Isabelle Bernard-Pierrot, Thierry Massfelder

**Affiliations:** ^1^ Department of Urology, New Civil Hospital and Fédération de Médecine Translationnelle de Strasbourg (FMTS), Strasbourg, France; ^2^ Urosphere, Toulouse, France; ^3^ Institut Curie, Centre National de la Recherche Scientifique (CNRS), UMR144, Molecular Oncology team, PSL Research University, Paris, France; ^4^ Sorbonne Universités, Université Pierre-et-Marie-Curie (UPMC), Univ Paris, Paris, France; ^5^ Department of Pathology, Institut Curie, Saint-Cloud, France; ^6^ Université de Versailles-Saint-Quentin-en-Yvelines (UVSQ), Paris-Saclay University, Versailles, France; ^7^ Inovarion, Paris, France; ^8^ La Ligue Contre Le Cancer, Paris, France; ^9^ Department of Urology, Zhongnan Hospital of Wuhan University, Wuhan, China; ^10^ Department of Urology, Rangueil Hospital, Toulouse, France; ^11^ Department of Genetics, Institut Curie, Paris, France; ^12^ Department of Pathology, New civil Hospital, Strasbourg, France; ^13^ INSERM (French National Institute of Health and Medical Research) UMR_S1260, Université de Strasbourg, Regenerative Nanomedicine, Centre de Recherche en Biomédecine de Strasbourg, Strasbourg, France

**Keywords:** urothelial carcinoma, squamous cell carcinoma, upper-urinary tract carcinoma, luminal tumors, basal tumors, tyrosine kinase receptor, molecular subtypes, tumor heterogeneity

## Abstract

**Background:**

Muscle-invasive bladder cancer (MIBC) and upper urinary tract urothelial carcinoma (UTUC) are molecularly heterogeneous. Despite chemotherapies, immunotherapies, or anti-fibroblast growth factor receptor (FGFR) treatments, these tumors are still of a poor outcome. Our objective was to develop a bank of patient-derived xenografts (PDXs) recapitulating the molecular heterogeneity of MIBC and UTUC, to facilitate the preclinical identification of therapies.

**Methods:**

Fresh tumors were obtained from patients and subcutaneously engrafted into immune-compromised mice. Patient tumors and matched PDXs were compared regarding histopathology, transcriptomic (microarrays), and genomic profiles [targeted Next-Generation Sequencing (NGS)]. Several PDXs were treated with chemotherapy (cisplatin/gemcitabine) or targeted therapies [FGFR and epidermal growth factor (EGFR) inhibitors].

**Results:**

A total of 31 PDXs were established from 1 non-MIBC, 25 MIBC, and 5 upper urinary tract tumors, including 28 urothelial (UC) and 3 squamous cell carcinomas (SCCs). Integrated genomic and transcriptomic profiling identified the PDXs of three different consensus molecular subtypes [basal/squamous (Ba/Sq), luminal papillary, and luminal unstable] and included *FGFR3*-mutated PDXs. High histological and genomic concordance was found between matched patient tumor/PDX. Discordance in molecular subtypes, such as a Ba/Sq patient tumor giving rise to a luminal papillary PDX, was observed (n=5) at molecular and histological levels. Ten models were treated with cisplatin-based chemotherapy, and we did not observe any association between subtypes and the response. Of the three Ba/Sq models treated with anti-EGFR therapy, two models were sensitive, and one model, of the sarcomatoid variant, was resistant. The treatment of three FGFR3-mutant PDXs with combined FGFR/EGFR inhibitors was more efficient than anti-FGFR3 treatment alone.

**Conclusions:**

We developed preclinical PDX models that recapitulate the molecular heterogeneity of MIBCs and UTUC, including actionable mutations, which will represent an essential tool in therapy development. The pharmacological characterization of the PDXs suggested that the upper urinary tract and MIBCs, not only UC but also SCC, with similar molecular characteristics could benefit from the same treatments including anti-FGFR for FGFR3-mutated tumors and anti-EGFR for basal ones and showed a benefit for combined FGFR/EGFR inhibition in FGFR3-mutant PDXs, compared to FGFR inhibition alone.

## Introduction

Bladder cancer (BCa) is the ninth most common cancer type worldwide with an estimated 549,000 new cases in 2018 (1). Histologically, 90%–95% of BCa are urothelial cell carcinomas (UCs) and 5% are squamous cell carcinomas (SCCs) (2). Although less frequent, UC may also develop in the upper urinary tract (2%–5% of UCs). Muscle-invasive BCas (MIBCs) are of very poor outcome, with an overall 5-year survival of 50%–60% and less than 10% for patients with localized disease or distant metastasis, respectively. Localized UC/SCC-MIBC and high-risk upper tract urothelial carcinoma (UTUC) are treated by radical cystectomy and radical nephroureterectomy, respectively, with the addition of adjuvant or neoadjuvant chemotherapies (2, 3). In advanced-setting UC, immune checkpoint or fibroblast growth factor receptor (FGFR) inhibitors (for tumors presenting *FGFR2/3* genetic alterations) may also be proposed (2, 4), while there is no standard-of-care treatment for SCC. Recently, two antibody–drug conjugates (ADCs) have shown efficacy in urothelial cancers, sacituzumab govitecan (5) (anti-TROP2) and enfortumab vedotin (6) (anti-Nectin4). Notably, the expression of NECTIN4 and TROP2 is variable across BCas, with NECTIN4 expression higher in luminal tumors (7, 8), which could impact the efficacy of these drugs. We also find in PDX transcriptomic analysis a heterogeneity of NECTIN4 and TROP2 expression, with a higher expression of NECTIN4 in luminal tumors. However, despite these treatments, the outcome remains poor, and the identification of new therapies is still needed. The development of relevant preclinical models is critical to reach this objective.

MIBCs constitute a heterogeneous group of tumors at the morphological and molecular levels. With the aim to improve the prediction of the clinical outcome and treatment response, an international consortium has recently reached a consensus molecular classification based on transcriptomic data, for MIBC, including six subtypes, facilitating interstudy comparisons (9). These subtypes can be divided into broad luminal (differentiated) and basal/squamous (Ba/Sq) groups. Although current systemic treatments are not based on molecular classification, some subtype features are associated with treatment response. For example, Ba/Sq tumors have worse prognosis than luminal-papillary (LumP) tumors [5], which commonly show the genetic alterations of FGFR3 (10). These subtypes have also been suggested to have a different sensitivity to chemotherapies, although results are currently inconsistent between studies (11–14). The Ba/Sq subtype and bladder SCC are associated with EGFR activation and sensitivity to anti-EGFR treatments in preclinical models (9, 15–18). UTUCs are molecularly comparable to MIBCs but have some distinct features, such as a higher prevalence of microsatellite instability (MSI) (19).

The molecular heterogeneity of MIBCs and UTUC and their divergent therapy sensitivities entails the need for a representative panel of preclinical models for unbiased therapeutic evaluation. Therapeutic testing in PDXs is highly effective in predicting the efficacy of both chemotherapies and targeted therapies (20, 21). Although few studies have reported the development of PDX models from MIBC, none have performed integrated genomic, transcriptomic, and pharmacological characterization of a bank of PDX reflecting the biological diversity of MIBC (22–24). Recently, a bank of 17 PDXs from UTUC has been reported (25) but no model exists for SCC, for which no standard of care is established.

In the present report, we describe the development and characterization of a bank of 31 PDXs, which maintain the characteristics of patient tumors and reflect the diversity of the molecular subtypes of bladder and upper urinary tract cancers. The evaluation of pharmacological responses to standard of care and targeted therapies suggests that non-sarcomatoid Ba/Sq tumors, notably SCC, could benefit from anti-EGFR therapies. In *FGFR3*-mutated tumor PDXs, an anti-FGFR and anti-EGFR combination therapy improves tumor response compared to FGFR inhibition alone.

## Materials and methods

### Animals

Four- to five-week-old immunodeficient mice (male) were purchased from Charles River Laboratories (L’Arbresle, France). Mice were maintained under specific pathogen-free conditions. Their care and housing were conducted in accordance with the European Community Council Directive 2010/63/UE and the French Ministry for Agriculture, Agrifood and Forestry Decree 2013-118. Experimental protocols were reviewed by CEEA-122 Ethical Committee for Protection of Animals used for Scientific Purposes and approved by French Ministry for National Education, Higher Education and Research under the number *APAFIS#14811-2018042316405732 v4*. The animal facility was maintained under artificial lighting (12 h) between 7:00 a.m. and 7:00 p.m. at a controlled ambient temperature of 22 ± 2°C and the relative humidity rate maintained at 55 ± 10%.

### Specimen acquisition

From January 2009 to October 2019, patient tumors and matched normal tissues were obtained from 153 patients treated for bladder or ureteral cancers undergoing surgery either at the Hospital of Strasbourg (France) (n=135) or the Hospital of Toulouse (France) (n=18) in accordance with all relevant guidelines and regulations. All patients provided written informed consent. The incoming material of every donor patient was anonymized by receiving a chronological unique number, subsequently used to identify the corresponding PDX model. The specimens were examined, sectioned, and selected by pathologists for histological analyses and xenografts. Clinical and demographic information were obtained prospectively.

### Patient-derived xenograft establishment

The PDX models of MIBC were generated by engrafting tumor tissues directly obtained from patients. Viable tumor tissue was macrodissected, and tumor pieces were then prepared for implantation. The NMRI nude (Rj : NMRI-Foxn1^nu/nu^) immunodeficient mice strain was used for tissue implantation. Grafts were implanted into the interscapular fat pad. When s.c. xenograft tumors reached ~1,000–1,500 mm^3^, they were serially transplanted for expansion into new mice. In addition, harvested xenograft material was cryopreserved for future implantations and/or fixed in 4% formalin for 24 h before paraffin embedding and/or stored at -80°C for subsequent analyses. A model was defined as established when stable growth over at least three passages and regrowth after a freeze–thaw cycle could be observed. The take rate (the proportion of mice developing tumors after the transplantation of the PDX) and passage time were recorded for every model and every individual passage. Tumor growth was determined weekly by a two-dimensional measurement with a caliper. The tumor volume was calculated as: TV (mm^3^) = [length (mm) × width (mm)^2^]*π/6, where the length and width are the longest and shortest diameters of the tumor, respectively. Animals were sacrificed when the tumor volume reached 2,000 mm^3^.

### Histopathology and immunohistochemistry

For all PDX models, primary and passaged tumors preserved in formalin for 24 h were paraffin-embedded, sectioned into 4-μm-thick cuts, and placed on glass slides. The analysis of hematoxylin and eosin (H&E)-stained slides was performed by two experienced uropathologists.

### RNA/DNA/protein extraction

Each frozen PDX fragment was ground to powder and subdivided for triple RNA, DNA, and protein extraction. RNA isolation was performed using TRIzol, while phenol/chloroform/isoamyl alcohol extraction was used for DNA isolation. See “Western blot” section for the protein extraction method.

### Real-time reverse transcription–quantitative PCR

Reverse transcription was performed on 1 µg of total RNA using a high-capacity cDNA reverse transcription kit (Thermo Fisher Scientific, Illkirch, France). cDNAs were amplified by PCR in a Roche real-time thermal cycler, with the Roche Taqman master mix (Roche) and Taqman probe/primer pairs as follows:

**Table:** 

Gene	Primer Forward	Primer Reverse
*EGFR*	GATCCAAGCTGTCCCAATG	GCACAGATGATTTTGGTCAGTT
*ERBB2*	CAACTGCACCCACTCCTGT	GCAGAGATGATGGACGTCAG
*ERBB3*	GGGAACCTTGAGATTGTGCT	CCTGTCACTTCTCGAATCCAC

### Sanger sequencing

The coding exons and splice junctions of *PPARG* were amplified from genomic DNA by PCR with gene-specific primers available on request and sequenced by the Sanger method as described (26).

### Weighted *in silico* pathology

WISP (Weighted *In Silico* Pathology; https://cit-bioinfo.github.io/WISP/) is an approach to assess intratumoral heterogeneity from bulk molecular profiles. Based on predefined pure molecular or histological populations for a particular cancer type, this approach gives a fine description of each tumor in a standardized way. The methodology is based on non-negative least squares regression and quadratic programming optimization for estimating the mixed proportions of distinct populations for a tumor sample. It can be applied on transcriptomic or methylation data. The output is the mixing proportion estimations for all samples.

For this analysis, we classified the samples from the MIBC CIT cohort (15) into a luminal or basal subtype according to the BASE47 classifier (27) to refine pure samples and calculate pure population centroid profiles, using standard parameters. Then, we estimated the mixed proportions of pure populations for each of our patient tumor and PDX samples, without scaling.

### Short tandem repeat signature

Patient tumors and corresponding PDX DNA samples were subjected to STR using the Authentifiler PCR amplification kit (Thermo Fisher Scientific, Illkirch, France) that amplifies nine unique STR loci and the amelogenin gender-determining marker, according to the manufacturer’s instructions. PCR products were separated by capillary electrophoresis on ABI PRISM 3100, and results were analyzed using the GeneMapper software.

### Genomic alterations detection: mutations, copy number variant detection, variant calling, and tumor mutational burden

Patient tumors and PDX were sequenced with a targeted NGS panel (called “DRAGON”) that has been developed by the genetics department of Institut Curie (Paris, France) and can detect mutations, copy number alterations (CNA), tumor mutational burden and microsatellite instability. It is composed of 571 genes of interest in oncology for diagnosis, prognosis, and theragnostics (**Supplementary Table 1**). NGS primers were selected based on their specificity on the human genome. The whole method is described in **Supplemental Method Dragon**.

Deleterious genomic alterations were defined as follows: (i) for oncogenes, only gain-of-function mutations were considered (i.e., hotspot missense mutations, in-frame insertions/deletions/splicing described as oncogenic), (ii) for tumor suppressor genes, only loss-of-function mutations were considered (i.e., biallelic truncating alterations—non-sense mutations, frameshift insertions/deletions/splicing—or monoallelic truncating alterations associated with heterozygous deletion detected by copy number analysis).

### Gene expression analysis

#### Gene expression arrays/transcriptomic data/consensus class

The RNA of 31 PDX and patient tumors samples were hybridized in three batches in Affymetrix Human Genome U133 plus 2.0 Array Plates (Santa Clara, CA) according to Affymetrix standard protocols. Raw CEL files were RMA-normalized (28) using R statistical software. PCA confirmed that no batch effect was observed. The arrays were mapped to genes with a Brainarray Custom CDF (Human EntrezG version 24) (29).

Molecular consensus classes were determined with the “consensusMIBC” R package (v1.1.0, https://github.com/cit-bioinfo/consensusMIBC) using the RMA-normalized transcriptomic data. For WISP, we used a previously published dataset (15), which contains human MIBC samples (n = 85) also hybridized with Affymetrix Human Genome U133 plus 2.0 according to Affymetrix standard protocols. The raw CEL files used here are available from ArrayExpress (http://www.ebi.ac.uk/arrayexpress/) under accession number E-MTAB-1803.

Raw CEL files were RMA-normalized using R statistical software. The arrays were mapped to genes with a Brainarray Custom CDF (Human EntrezG version 23) (29)

In both datasets, we obtained a log2-transformed expression matrix with one value per gene.

### Regulatory networks

The regulatory network was reverse-engineered by ARACNe-AP (30) from human urothelial cancer tissue datasets profiled by RNA-Seq from TCGA (n=414). The RNA-seq data was downloaded from the TCGA data portal using the TCGAbiolinks package (R). Raw counts were normalized to account for different library sizes, and the variance was stabilized with VST function in the DESeq2 R-package (31)

ARACNe was run with 100 bootstrap iterations using all probe-clusters mapping to a set of 1,740 transcription factors. The parameters used were standard parameters, with the mutual information p-value threshold of 10^−8^.

### Regulon activity—VIPER

The VIPER algorithm tests for regulon enrichment based on gene expression signatures (32), using the regulatory network obtained from ARACNe on urothelial cancer, and we computed the enrichment of each regulon on the gene expression signature using different implementations of the analytic rank-based enrichment analysis algorithm.

### Dual-staining immunohistochemistry

Dual immunostaining for GATA3 and KRT5/6 on Formalin-Fixed, Paraffin-Embedded (FFPE) samples was performed to screen for homogeneous or heterogeneous luminal or Ba/Sq tumors at the immunohistochemical level. Automated sequential dual-staining immunohistochemistry (IHC; Discovery, Roche/Ventana, Tucson, AZ, USA) was used according to the manufacturer’s instructions. Tissue sections cut at 3 µm were dewaxed and subjected to antigen retrieval, then incubated first with a GATA3-specific rabbit monoclonal antibody (1:300, clone ZR65 Diagomics, Blagnac, France), followed by an HRP-conjugated anti-rabbit IgG secondary antibody (MP-7401, Vector). The antigen–antibody reaction was detected using an ImmPACT DAB reagent (SK-4105, Vector), producing brown staining in positive nuclei. In the second sequence, a primary rabbit monoclonal antibody against KRT5/6 (1:100, clone EP24/EP67; Diagomics, Blagnac, France) was incubated, followed by alkaline phosphatase–conjugated anti-rabbit IgG secondary antibody (ENZ-ACC110-0150, Enzo). The antigen–antibody reaction was revealed using ImmPACT red reagent (Vector), producing red staining in positive cytoplasm. A normal urothelium was used as a positive control. The staining for GATA3 and KRT5/6 was evaluated by one blinded pathologist (JF), providing respective quick scores (QSs) calculated as intensity (0–3) multiplied by the percentage of stained tumor cells and normalized to [0;1]. Immunohistochemical thresholds for Ba/Sq tumors (IHC-Ba/Sq) defined as QS(KRT5/6) >0.14 and QS(GATA3) <0.02, or luminal QS(GATA3) >0.14 and QS(KRT5/6) <0.02, were used (33). Tumors showing IHC-Ba/Sq and non-Ba/Sq areas were defined as having intratumoral heterogeneity.

### *In vivo* efficacy studies

Erlotinib (EGFR inhibitor) was purchased from MedChemExpress (Sollentuna, Sweden) and administered orally (gavage) 5 days per week during 4 weeks at a dose of 30 or 90 mg/kg (0.5% carboxymethylcellulose in PBS).

Erdafitinib (pan-FGFR inhibitor) was purchased from MedChemExpress (Sollentuna, Sweden) and administered orally (gavage) 6 days per week during 4 weeks at a dose of 10 or 30 mg/kg (20% 2-hydroxypropyl β-cyclodextrin in distilled water).

BGJ398 (pan-FGFR inhibitor) was purchased from LC Laboratories and administered orally (gavage) 6 days per week during 4 weeks at a dose of 30 mg/kg.

Cisplatin and gemcitabine were purchased from Sigma (St. Quentin Fallavier, France). Both drugs were administered intraperitoneally at a dose of 60 mg/kg (NaCl 0.9%) once a week (D0, D7, D14) and 4 mg/kg (NaCl 0.9%) once every 3 weeks (D1), respectively.

For efficacy studies, mice were implanted as described above. Tumor fragments were transplanted into 6-week-old NMRI mice. When the tumor reached a volume comprised between 60 and 270 mm^3^, mice were randomly assigned to the vehicle or treatment groups (n= 7–10). The tumor volume was calculated as: TV (mm^3^) = [length (mm) × width (mm)^2^]*π/6, where the length and width are the longest and shortest diameters of the tumor, respectively. Tumor volumes were then reported to the initial volume as relative tumor volume (RTV). The means of the RTV in the same treatment group were calculated. Growth curves were generated using the GraphPad Prism software.

### Western blot

Frozen PDX samples were resuspended in Laemmli lysis buffer [50-mM Tris‐HCl (pH 6.8), 2-mM DTT, 2.5-mM EDTA, 2.5-mM EGTA, 2% SDS, 5% glycerol with protease inhibitors and phosphatase inhibitors (Roche)], and the resulting lysates were clarified by centrifugation. The protein concentration of the supernatants was determined with the BCA protein assay (Thermo Scientific, Illkirch, France). Proteins (10–50 μg) were resolved by SDS-PAGE in 10% polyacrylamide gels, electrotransferred onto Bio-Rad nitrocellulose membranes, and analyzed with antibodies against β-actin (Sigma-Aldrich #A2228, used at 1/25,000), or the extracellular domain of FGFR3 (Abcam, # ab133644, 1/5,000). Anti-mouse IgG, HRP-linked, and anti-rabbit IgG, HRP-linked antibody (Cell Signaling Technology # 7076 and # 7074, used at 1/3,000, Saint-Cyr-L’École, France) were used as secondary antibodies. Protein loading was checked by the Amido Black staining of the membrane after electrotransfer.

### Statistical and bioinformatic analysis

Comparisons between PDX treatment responses were done using the Mann–Whitney test.

Bioinformatics analyses was performed with R (4.0.2).

## Results

### Establishment of urothelial PDXs

We successfully obtained 31 PDXs from 153 tumors (global engraftment rate ~20%, median latency 27.5 days with a range of 10–70 days) from January 2009 to October 2019. The main clinicopathological characteristics of the patients and patient tumors are summarized in **Table 1** (additional clinical data from patients are presented in **Supplementary Table 2**). As expected, the majority of PDXs was derived from male patients (84%), with bladder as the primary site (84%) and were urothelial carcinomas (90%). The frequency of squamous cell carcinomas (SCC, 3/31, 10%) was higher than the known frequency of these tumors, potentially owing to a higher engraftment success rate (n=4/10, with one of the four that could not be further analyzed due to patient serology). One PDX was derived from a non-muscle-invasive BCa (NMIBC). Four patients had received systemic or radiotherapy treatment before the establishment of the PDX.

**Table 1:** Clinicopathological characteristics of the samples used for patient-derived xenograft.

Variables	All patients
**Total**	31
**Median age, years (IQR)**	75 (52–88)
**Gender**	
Male	26 (84%)
Female	5 (16%)
**Primary site**	
Bladder	26 (84%)
Upper tract	5 (16%)
**Histology**	
**Urothelial**	28* (90%)
Conventional	17
Squamous differentiation	7
Sarcomatoid	2
Plasmacytoid	1
Glandular differentiation	1
Micropapillary	1
Poorly differentiated	5
**Squamous**	3 (10%)
**Prior Treatment**	
No	19 (61%)
BCG	3 (10%)
Cisplatin-based CT	3 (10%)
Radiotherapy + cisplatin-based CT	1 (3%)
NA	5 (16%)
**pT Stage**	
<pT2 (non-MIBC)	1 (3%)
≥pT2	30 (97%)
**pN Stage**	
pN0	7 (23%)
pN+	10 (32%)
pNx	14 (45%)
**Associated CIS**	
Yes	4 (13%)
No	27 (87%
**Relapse (Yes)**	4 (13%)

CT, chemotherapy.

*Variant total is above n=31 as some tumors have multiple variants.

### Histological and genomic characterization of PDXs

**Figure 1:**
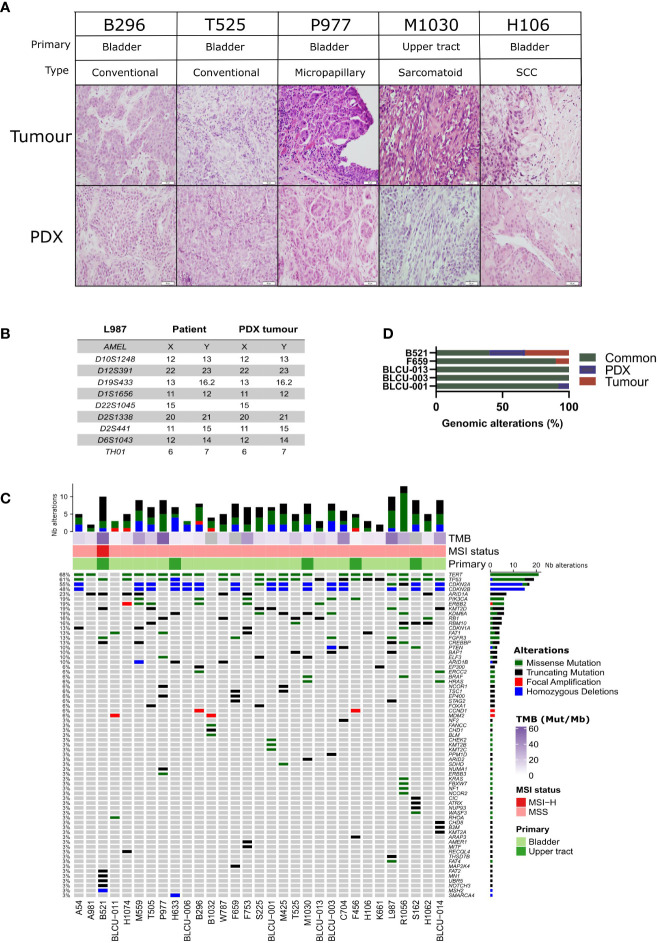
Histological and genomic characteristics of patient tumors and paired derived patient-derived xenografts (PDXs). **(A)** Histology of bladder/ureteral patient tumors and corresponding PDX models demonstrated similar histological features as assessed by hematoxylin and eosin (H&E) staining. Scale bar corresponds to 50 µm. H&E slides of a section of each patient tumor and matching PDX were reviewed by a board-certified pathologist, and representative pictures for the different histologies or variants are shown. **(B)** Short tandem repeat signature of a patient specimen and PDX tumor, an example of the L987 case. **(C)** Somatic genomic landscape of 31 bladder and ureteral PDXs analyzed using an in-house targeted sequencing assay (571 cancer-related genes, **Supplementary Tables 2**, **3**), the tumor mutational burden per megabase (TMB, indicated in the log2 scale for each sample), and microsatellite instability–high (MSI-H) versus a microsatellite-stable (MSS) status. **(D)** Concordance of genomic alterations in five pairs tumor/PDX.

A histopathological analysis of the H&E-stained slides of patient tumors and PDXs was performed by two pathologists (VL, JF), based on the current WHO Classification of Tumors of the Urinary System (34). High histological concordance between patient tumors and PDXs was observed **(** **Figure 1A** **)** with the exception of three PDXs showing distinct histology compared to the matched patient tumors (M1030 and BLCU-011 lost the variant observed in the tumor, while a more squamous variant was observed in R1056, **Supplementary Table 2**). We confirmed by short tandem repeat (STR) profiling the concordant genetic identity between patient tumor and derived PDXs (**Supplementary Table 3** **)**, with 85%–100% of conserved STR for the different models, as an example for L987 in **Figure 1B**. We characterized genomic alterations for 571 cancer-related genes (**Supplementary Table 1** and **Supplementary Table 4**) in all 31 PDXs using a targeted next-generation sequencing assay, allowing the detection of mutations, the estimation of copy number alterations (CNA), tumor mutational burden (TMB), and MSI status (**Figure 1C** **).** As anticipated, the most frequent genomic alterations were mutations in *TERT* (68%) and *TP53* (61%) and the homozygous deletion of *CDKN2A/B* (~50%). We also identified activating mutations in potentially actionable genes such as *PIK3CA* (19%), *ERBB2* (19%), *FGFR3* (13%), *BRAF* (6%), *ERBB3* (3%), *KRAS G12C* (3%), and truncating mutations in the epigenetic genes *KDM6A* (19%), *ARID1A* (23%), *KMT2D (*19%), *KMT2A/B/C* (3% each), and *ARID2* (3%). One UTUC PDX displayed microsatellite instability associated with a bi-allelic deletion of *MSH2*, and this alteration was also observed in the parental tumor. To determine whether the PDXs retain the genomic alterations of the matched patient tumor, we sequenced five patient tumor/PDX pairs. The overall concordance of observed genomic alterations was high (90%–100%) (**Figure 1D**), except for the MSI-high PDX (B521) that harbored high TMB (40%). PPARG pathway activation, through PPARG amplifications or RXRA- and PPARG-activating mutations, is a known key feature of luminal tumors and a potential therapeutic target (16, 26, 35–37). We did not observe any *RXRA* mutation in our sequencing analyses. Since *PPARG* was not in our targeted panel, we performed a Sanger sequencing of the hotspot region within the ligand-binding domain of *PPARG* (26) and identified one patient tumor/PDX pair (B521) harboring the T475M-activating mutation (n=1/23 tested PDX) and one patient tumor/PDX pair (M559) harboring the non-characterized and non-recurrent L339F mutation (26).

### PDXs recapitulate the molecular subtypes and intratumoral heterogeneity

**Figure 2:**
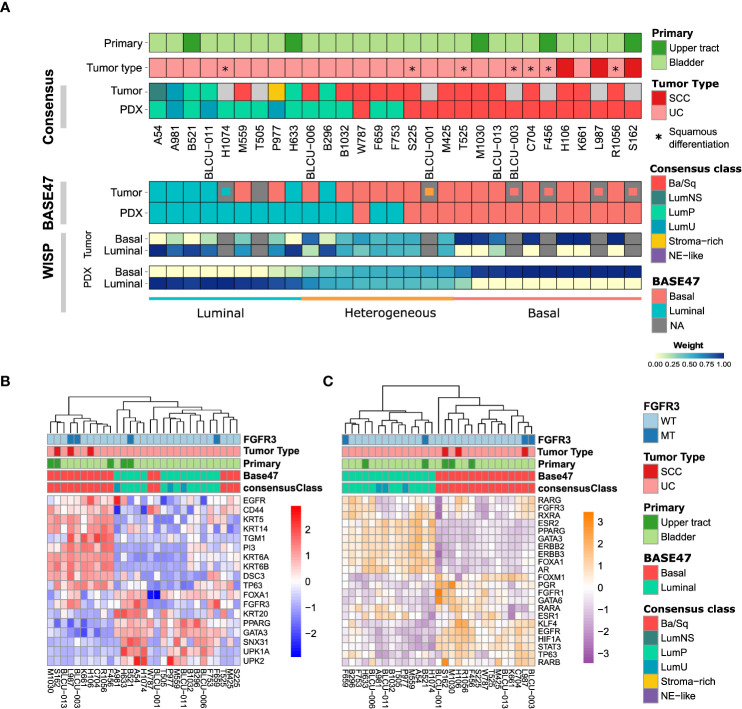
Transcriptomic analysis of patient tumors and paired PDXs. **(A)** Tumors and PDXs were classified into six subtypes using transcriptomic Affymetrix U133plus2 array data according to the molecular consensus classifier developed for MIBC, the corresponding box colors indicated in the legend on the right. A total of 22 patient tumor–PDX pairs and 7 individual PDXs were analyzed, where tumors without transcriptomic data are indicated in gray. Urothelial carcinomas with divergent squamous differentiation are highlighted with *. **(B)** Upper panels: tumors and PDXs were classified using transcriptomic data as luminal and basal subtypes according to the BASE47 classifier (27). In patient tumors with missing transcriptomic data, we assessed the luminal (blue), basal (red), or heterogeneous (orange) subtype by immunohistochemistry (IHC; inset small boxes), as defined in methods. Lower panels: the intra-tumoral heterogeneity and proportion of luminal and basal subtype admixture as evaluated from transcriptomic profiles using the WISP (Weighted *In Silico* Pathology) algorithm. Based on the PDX WISP results, samples were molecularly classified as luminal, basal, or heterogeneous. **(B)** Heatmap of PDX samples based on the gene expression of selected luminal or basal markers. **(C)** Heatmap of PDX samples based on the regulon activity of the main regulators previously identified within the different molecular subtypes of MIBC (9, 10).

We then sought to determine whether PDX models recapitulate the molecular subtypes of patient tumors. In total, transcriptomic data using Affymetrix U133plus2 array were available for 22 patient tumor/PDX pairs and 8 individual PDXs. Unsupervised clustering analysis using the top 200 most variant genes did not highlight any segregation in PDXs by the primary site or histological tumor (**Supplementary Figure 1**, with the exclusion of the NMIBC PDX for the transcriptomic subtype analysis). We therefore considered all patient tumors/PDX similarly and stratified them into six subtypes by applying the molecular consensus classifier (9) (**Figure 2A**). We observed 15 Ba/Sq (52%), 11 LumP (38%), and 3 luminal-unstable (LumU) (10%) PDXs. All three SCC patient tumors/PDXs were classified as Ba/Sq. In contrast to genomic and histological characteristics, transcriptomic profiles were less stable between patient tumor/PDX pairs. Specifically, eight patient tumors gave rise to a PDX with a distinct molecular subtype (36%) including five Ba/Sq patient tumors to LumP PDX, one LumP to LumU, one stroma-rich to LumU, and one LumNS to the LumP subtype. We also stratified patient tumors and PDXs according to the BASE47 classifier (27) (**Figure 2A**). Among the 16/22 patient tumors that were classified as basal, 6 gave rise to a luminal PDX. In contrast, all luminal tumors formed luminal PDXs. For six matched tumor-PDX pairs, for which we did not have transcriptomic data, we performed GATA3 (luminal) and KRT5/6 (basal) dual immunostaining to assign molecular subtypes using previously defined thresholds for each marker (33) and we did not observe a difference in the subtype between tumors and PDXs (**Figure 2A**). To explore whether the basal- to-luminal discordance could be related to intratumoral heterogeneity, we evaluated the molecular heterogeneity in both patient tumors and PDXs using the WISP algorithm (**Figure 2A**). We observed the admixed proportions of luminal and basal subtypes in 59.1% of patient tumors and 41.4% of PDXs, including all six cases with discordant BASE47 subtypes. The high molecular heterogeneity found in the six basal tumors that gave rise to luminal PDXs was conserved in most of the matched PDXs. These findings suggest an intrinsic plasticity of these tumors, leading to a shift in subtype rather than a sampling bias of an area of a given subtype within a molecularly heterogeneous patient tumor (**Figure 2A**, lower panels).

Using defined biomarkers of luminal and basal differentiation (9, 10), we confirmed that PDXs were globally separated between luminal, differentiated tumors and basal tumors (**Figure 2B** and **Supplementary Figure 1**). Concerning NECTIN4 and TROP2, which could be targeted by drug-conjugated antibodies (5, 6), we also found in the PDX transcriptomic analysis a heterogeneity of NECTIN4 and TROP2 expression, with a higher expression of NECTIN4 in luminal tumors (**Supplementary Figure 2**).

To further explore the transcriptomes of our PDXs, we inferred the activity of 22 major regulons, as defined in TCGA analysis (10) (**Figure 2C**). Using this approach, we observed a perfect separation of luminal and basal PDXs in clustering analysis, independently of the primary site of patient tumor. Similar to patient tumors from TCGA, we observed a higher *EGFR* regulon activity in basal PDXs and a higher *PPARG/ERBB2/ERBB3* regulon activity in luminal PDXs (10). Of note, *FGFR3* mutations are enriched in LumP tumors (9) and we observed *FGFR3* mutations not only in two LumP PDXs, including one derived from UTUC, but also in two Ba/Sq PDXs, including one derived from an SCC tumor (**Figures 2B**, **C**) (9). As expected, high FGFR3 regulon activity was observed in tumors bearing *FGFR3* mutations.

**Figure 3:**
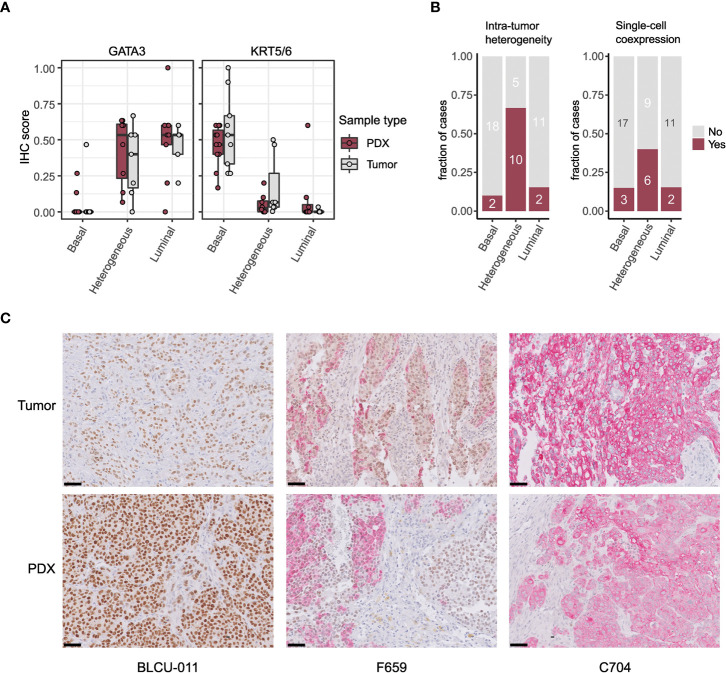
Intratumor heterogeneity in tumors and PDXs at the protein level by IHC. **(A)** GATA3 and KRT5/6 expression levels (normalized quick scores), grouped according to the PDX WISP molecular classification (luminal, heterogeneous, and Ba/Sq, **Figure 2A**). **(B)** Proportion of tumors with intratumoral heterogeneity (left) and GATA3 + KRT5/6 coexpression at the single-cell level (right), grouped according to the PDX WISP molecular classification (luminal, heterogeneous, and Ba/Sq, **Figure 2A**). **(C)** Patterns of dual IHC staining for GATA3 (brown, nuclear) and KRT5/6 (red, cytoplasmic) in the paired tumor/PDX of a luminal (BLCU-011), a heterogeneous (F659), and a Ba/Sq (C704) example.

To validate the intratumoral molecular subtype admixture inferred from the transcriptomic data *in situ*, we performed dual IHC staining combining a luminal (GATA3) and a basal marker (KRT5/6) in a subset of patient tumors and PDXs. We confirmed the presence of a subtype marker expression admixture *in situ*, with either spatially distinct areas showing different IHC profiles (defined hereafter as intratumoral heterogeneity) or a single-cell coexpression of the two markers, or a combination of both patterns of admixture. By comparing the results between tumors/PDXs classified as basal, luminal, or heterogenous based on the WISP analysis (**Figures 2A**, **3**), we observed that tumors/PDXs classified as pure luminal or Ba/Sq based on transcriptomic data displayed higher GATA3 or KRT5/6 staining levels, respectively (**Figure 3A**). The WISP heterogeneous samples had more intermediate staining levels of both GATA3 and KRT5/6 (**Figure 3A**) and showed more intratumoral heterogeneity compared to pure Ba/Sq or luminal cases (10/15 vs. 4/29, p<0.01) (**Figure 3B**). Among samples with intratumoral heterogeneity, a GATA3-KRT5/6 coexpression at the single-cell level was also identified in 9/14 samples (**Figures 3B, C**).

### Chemosensitivity of patient-derived xenografts

**Figure 4:**
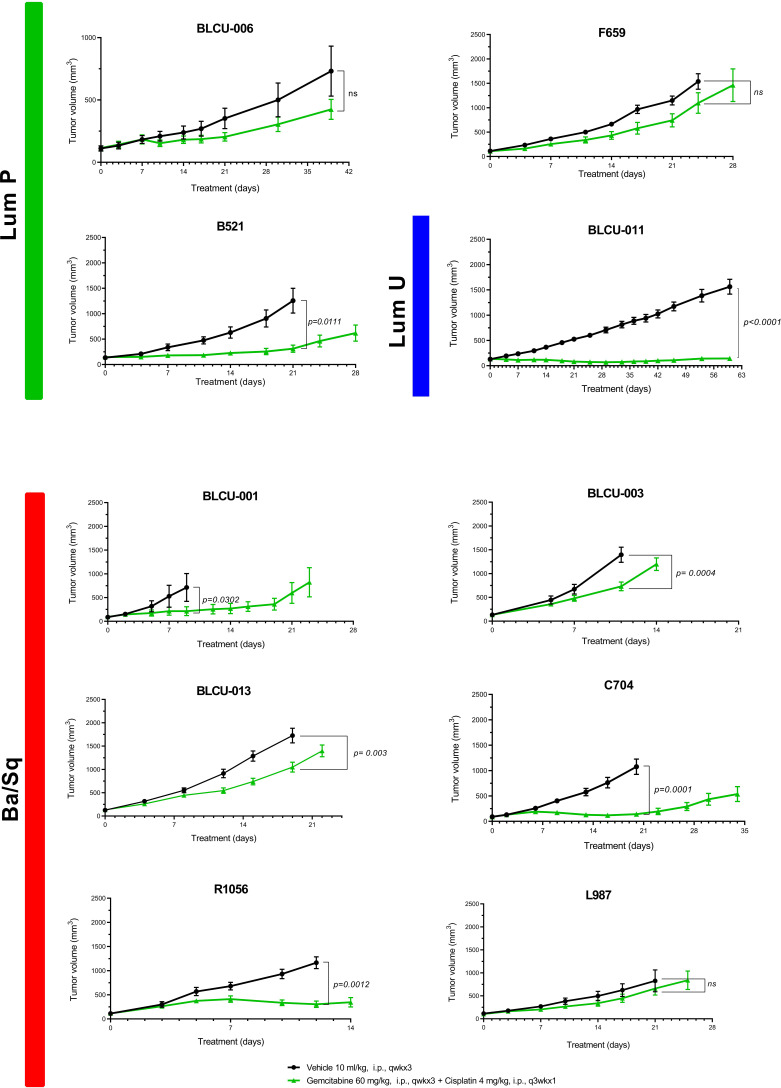
Chemosensitivity of representative basal and luminal PDX models. Mice with established PDXs (67–270 mm^3^) were treated with cisplatin plus gemcitabine (green) as indicated, and control mice were treated with vehicle alone (black) (n = 7–10 animals per group). Tumor size was measured at the indicated time points. Data are presented as mean ± SEM. Results were compared using the Mann–Whitney test. n.s, non-significant.

With cisplatin-based chemotherapy being the standard of care of MIBC and high-risk UTUC, we assessed the sensitivity of 10 PDXs representative of the different subtypes (six basal, four luminal) to cisplatin plus gemcitabine (**Figure 4**). We did not observe a significant difference between the proportion of basal (5/6, 83%) and luminal PDXs (2/4, 50%) with significant growth inhibition upon treatment (Fisher’s exact test p=0.5). Due to the low number of recurrent genomic alterations and the number of models tested, it was not possible to statistically explore the association between genomic alterations and chemosensitivity.

### EGFR-targeted therapy is effective in non-sarcomatoid basal/squamous patient-derived xenografts

**Figure 5:**
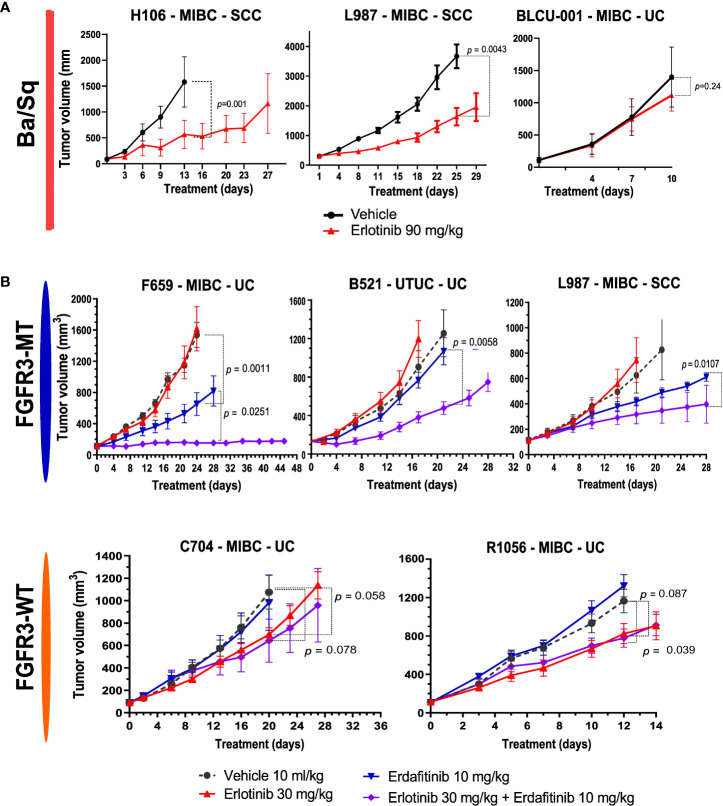
Sensitivity to anti-EGFR or the combination of EGFR and FGFR inhibitors in Ba/Sq and *FGFR3*-mutated PDXs. **(A)** Mice with established basal/squamous PDXs (67–270 mm^3^) were treated with anti-EGFR (erlotinib 90 mg/kg, red) or control vehicle alone (black). **(B)** Mice with established *FGFR3*-mutated tumors and controls were treated with control vehicle (black), low-dose anti-EGFR (erlotinib 30 mg/kg, red), a pan-FGFR inhibitor (erdafitinib 10 mg/kg, blue), or the combination (purple), as indicated (n = 7–10 animals per group). Tumor size was measured at the indicated time points. Data are presented as mean ± SEM. Results were compared using the Mann–Whitney test.

In relation to the high EGFR activity in basal tumors, we have previously shown that EGFR is an effective therapeutic target in different *in vivo* basal preclinical models (15). The aggressive sarcomatoid variant of MIBC is suggested to occur through the progression of Ba/Sq tumors (38). We recently analyzed sarcomatoid tumor transcriptomes and identified a loss of EGFR regulon activity during the progression of Ba/Sq tumors to the sarcomatoid variant (Fontugne et al., unpublished results). In line with these findings, we identified a low EGFR regulon activity in a PDX (BLCU-001) compared to the other Ba/Sq PDXs, which was classified as a Ba/Sq sarcomatoid tumor (**Figure 2C** and **Supplementary Table 1**). To assess whether the loss of EGFR activity could impact the sensitivity to EGFR inhibition, we compared the effect of anti-EGFR treatment (erlotinib) in two Ba/Sq models presenting high EGFR-regulon activity (L987 and H106, **Figure 2C**) and BLCU-001 (**Figure 5A**). As expected, the two SCC Ba/Sq models were sensitive to erlotinib whereas the Ba/Sq model with sarcomatoid differentiation was resistant (**Figure 5A**). Of note, two other Ba/Sq PDXs (C704 and R1056) were sensitive even when we used a lower dose of erdafitinib in a second experiment (**Figure 5B**).

### Combined inhibition of FGFR and EGFR improves response of *FGFR3*-mutated patient-derived xenograft compared to FGFR inhibition alone

FGFR inhibitors have already demonstrated clinical efficacy in FGFR3-altered tumors (4). However, resistance to treatment is systematically observed over time. Consistently, the treatment of two *FGFR3*-mutated PDXs, one Ba/Sq and one LumP, both presenting high expression levels of FGFR3 protein compared to other non-mutated PDXs, with a pan-FGFR-inhibitor (BGJ398) (**Supplementary Figure 3A**) only reduced tumor growth (**Supplementary Figure 3B**). Different preclinical studies using bladder-derived cell lines identified EGFR activation as a mechanism of resistance to FGFR inhibition (39–41). Using reverse transcription–quantitative PCR (RT-qPCR), we confirmed that this mechanism could be relevant in our PDX models since anti-FGFR treatment not only induced the overexpression of EGFR but also ERBB2 and ERBB3 in our *FGFR3*-mutated L987 model (**Supplementary Figure 3C**).

Therefore, we then tested if a combination of FGFR3 and EGFR/ERBB2 inhibition could overcome the compensatory upregulation of EGFR signaling and increase the sensitivity of FGFR3-mutated PDXs to FGFR inhibition, as previously observed for RT112 xenografts harboring FGFR3-TACC3 fusion (39). We treated five PDXs (three *FGFR3*-mutated: L987, B521, F659; two Ba/Sq *FGFR3*-WT: R1056, C704) with a pan-FGFR inhibitor (erdafitinib) that recently obtained FDA approval for locally advanced or metastatic MIBC with *FGFR3* genomic alterations and an EGFR inhibitor [erlotinib, which has been described to have an ERBB2 inhibitory effect (42)] alone and in combination (**Figure 5B**). To limit toxicity, we used suboptimal doses for each inhibitor when administered in combination. In these conditions, monotherapies presented limited effects, with significant growth inhibition in two *FGFR3*-mutated and in two basal PDXs upon erdafitinib and erlotinib treatment, respectively. The combination of erlotinib plus erdafitinib led to an improved response in all *FGFR3*-mutated PDXs compared to each of the monotherapies, while no additional benefit was observed for the combination in the Ba/Sq *FGFR3*-WT PDXs (**Figure 5B**).

## Discussion

The development of relevant preclinical models closely mimicking patient tumors is key in the identification of new potential therapies for precision medicine, and PDXs are widely used in this regard in oncology (24, 43).

We report here, like others (22–25), that PDXs derived from bladder and ureteral cancers preserved the histological and genomic properties of patient tumors. However, their transcriptomic profiles were less stable. Namely, we identified basal patient tumors giving rise to luminal PDXs. For these cases, both tumors and PDXs revealed high heterogeneity, suggesting intrinsic cell plasticity. Such heterogeneity and cell lineage plasticity were recently demonstrated by Sfakianos et al. in the basal N-butyl-N-(4-hydroxybutyl)-nitrosamine (BBN) chemically induced mouse bladder tumors using both single-cell transcriptomic analysis and Fluorescence-Activated Cell Sorting (FACS) analysis after the *in vivo* transplantation of FACS presorted and cultured tumor cells (44). Basal tumors present a more abundant stroma compared to luminal tumors including high infiltration by immune cells (9). Our results also suggest that, because the stroma in immune-deficient mice differs from human tumor stroma, the crosstalk between the tumor and stroma may impact the tumor cell phenotype. Such crosstalk has been reported in three-dimensional *ex vivo* models, where the absence of cancer-associated fibroblasts induced a shift from luminal tumors to basal organoids (45, 46) whereas basal organoids engrafted in mice then developed a luminal phenotype (45). Interestingly, the molecular heterogeneity of luminal/basal markers observed in tumor and PDX pairs also reinforces the relevance of our models in drug efficacy evaluation, given that tumor heterogeneity is a known cause of resistance to treatment (47).

We described potential actionable genomic alterations in our PDXs, such as *PIK3CA* mutation, *ERBB2* mutation/amplification, or *MDM2*-amplification (48, 49), whose frequencies are comparable with TCGA (10). Epigenetic drugs could also represent a promising therapeutic approach in monotherapy or in combination when considering the high proportions of PDXs harboring at least one mutation in epigenetic genes (50). Our models could thus be useful to evaluate such new potential therapies.

Previously, we proposed EGFR as a therapeutic target in basal MIBC based on findings from different *in vitro* and *in vivo* preclinical models (15). Here, we further validated the EGFR dependency of Ba/Sq tumors using PDX models. However, anti-EGFR treatments have shown disappointing results in the clinic, although some pathological responses have been observed in the neoadjuvant setting (51). This discrepancy between preclinical and clinical settings warrants further investigation. We hypothesize that the rich stroma of basal tumors contributes to anti-EGFR resistance, as recently demonstrated with cancer-associated fibroblasts in lung cancer (52). Indeed, rich stroma is absent in our preclinical models, with the exception of the BBN model, which presented only a moderate sensitivity to anti-EGFR treatment (15). Our results also suggest that sarcomatoid differentiation could be another mechanism of resistance to anti-EGFR treatment. In agreement, sarcomatoid differentiation is linked to EMT, which was previously shown to impair anti-EGFR sensitivity in lung cancer (53).

In agreement with previous studies (54, 55), we found that upper urinary tract and bladder urothelial carcinomas harbored similar genomic alterations but at different frequencies, except for the presence of MSI-H in one UTUC (54, 55). The diversity of our bank—including both MIBC [urothelial (UC) and squamous (SCC)] and UTUC—allowed us to observe that urothelial carcinoma PDXs originating from the upper urinary tract or bladder were also highly similar at the transcriptomic level. Additionally, we found that SCC samples did not classify into a molecularly distinct group of tumors, instead grouping with urothelial Ba/Sq carcinomas. Whereas up to now, SCC, UC, and UTUC were considered as highly different entities in terms of response to treatment, we found that the same molecular classifications/genetic alterations can predict the same therapeutic response for the three entities. Indeed, independently of the cell of origin, basal PDXs were sensitive to anti-EGFR therapies, except for sarcomatoid tumors, which are characterized by low EGFR activity. Furthermore, most FGFR3-mutated PDXs were sensitive to FGFR inhibition independently of their cell of origin or their luminal/basal phenotype.

Finally, it has been shown in RT112 xenografts (BCa cell line presenting an FGFR3-TACC3 fusion observed in 3% of MIBC) that a combination of anti-EGFR/anti-FGFR was more potent than anti-FGFR alone (39). We validated here these results using our more clinically relevant PDX models with *FGFR3* mutations (observed in 15% of MIBC) (three different models derived from UTUC, MIBC-UC, and MIBC-SCC) reinforcing the interest to further test this combination treatment in the clinics. For treatment optimization and to limit side effects and toxicity, it will be of interest to explore if this effect is more specifically associated with EGFR or HER2 inhibition.

## Conclusion

Muscle-invasive bladder and upper urinary tract cancers are heterogeneous and aggressive diseases with no satisfactory treatment. We have developed and characterized highly relevant preclinical models for bladder and upper tract carcinoma, recapitulating the molecular heterogeneity and drug responses observed in the clinic. Our work supports a benefit of combined FGFR and EGFR inhibition in FGFR3-mutated tumors. Overall, our models represent an essential tool for the development of new efficient therapies against these aggressive cancer types.

## Data availability statement

The datasets presented in this study can be found in online repositories. The names of the repository/repositories and accession number(s) can be found below: https://www.ncbi.nlm.nih.gov/geo/, GSE181962.

## Ethics statement

This study was reviewed and approved by Comité de Protection des Personnes Sud-Ouest et Outre-mer, DC 2019-3565]. Written informed consent was obtained from all participants for their participation in this study. The animal study was reviewed and approved by CEEA-122.

## Author contributions

IB-P had full access to all the data in the study and takes responsibility for the integrity of the data and the accuracy of the data analysis. HL, CB, ML and LC designed, performed experiments or bioinformatics analyses, analyzed and interpreted the data. HL, CB and ML developed the PDXs models. HL, XG, PR provided samples and helped with clinical data analysis. CB and ML performed the pharmacological characterization of the PDXs. CB, CK and JF prepared DNA and RNA for genomics analysis. CB performed STR analysis under TM supervision. CK performed and analyzed sanger sequencing. CK and VD performed immunohistochemical experiments under YA supervision. JF performed histo- and immunohistopathological analysis and designed pharmacological studies for sarcomatoid tumors under YA supervision. FD performed Western blot and RT-qPCR analysis. LC performed most of the bioinformatics analyses together with help of CSG, XM and AK for tumor’s/PDXs’ classification and for regulon analysis. ADR supervised AK and CG. EC normalized and annotated Affymetrix array data and centralized the data for bioinformatics analyses. JM-P and RB performed and analyzed targeted NGS under the supervision of IB. VL performed and supervised histopathological analysis of tumors and PDXs. YM and PL designed the study and supervised establishment of part of the PDXs and pharmacological characterization of PDXs. FR supervised the genomic analyses. TM and HL designed and supervised the establishment of part of the PDXs. IB-P and TM designed and supervised the research, analyzed and interpreted the data. LC, CB, JF, TM and IB-P wrote the paper. All authors contributed to the article and approved the submitted version.

## Funding

This work was supported by a grant from Ligue Nationale Contre le Cancer (LC, FD, JF, CK, CG, XM, YA, FR, IB-P) as an associated team (Equipe labellisee’), the “Carte d’Identité des Tumeurs” program initiated, developed, and funded by Ligue Nationale Contre le Cancer. This work was supported by the Institut National du Cancer: PRTK project “BoBCaT”. LC was supported by FRM (Fondation Recherche Médicale) and JF by the Fondation ARC pour la recherche sur le cancer. XM was supported by a fellowship from ITMO Cancer AVIESAN, within the framework of Cancer Plan. We also thank BPI France, the Region Alsace, the European Funds for Regional Development, the Strasbourg Urban Community, INSERM, and the University of Strasbourg for financial support (recipients TM and HL for the innovative Urolead SAS company that was acquired by Urosphere in 2017 and Urosphere supported by the European Funds for Regional Development).

## Acknowledgments

We thank David Gentien from the genomics platform of Institut Curie. We also thank the Alsace incubator SEMIA, Strasbourg, France, for advices and financial support (recipients TM and HL for Urolead SAS). We also thank the competitivity pole Alsace Biovalley, Illkirch-Graffenstaden, France for advices and labelization (TM and HL, for Urolead SAS). Finally, we also thank Alsace Satt Conectus, Illkirch-Graffenstaden, France, for technology transfer and advices (TM and HL, for Urolead SAS).

## Conflict of interest

Author’s CB, ML, YM and PL were employed by Urosphere and author FD was employed by Inovarion, Institut Curie, Strasbourg University and Urosphere have a collaboration contract for the transcriptomic, genomic, and pharmacological characterization of the PDX.

The remaining authors declare that the research was conducted in the absence of any commercial or financial relationships that could be construed as a potential conflict of interest.

## Publisher’s note

All claims expressed in this article are solely those of the authors and do not necessarily represent those of their affiliated organizations, or those of the publisher, the editors and the reviewers. Any product that may be evaluated in this article, or claim that may be made by its manufacturer, is not guaranteed or endorsed by the publisher.

## Supplementary material

Supplementary Figure 1 Unsupervised hierarchical clustering and heatmap of PDX samples based on genes with the most variant expression (n=200). PDX identifiers indicated at the bottom. Classifications indicated according to legend on the right (as in **Figure 2**).

Supplementary Figure 2 Expression of NECTIN4 and TROP2 (*TACSTD2*) in PDXs. **(A)** Heatmap of PDX samples based on *NECTIN4* and *TACSTD2* gene expression. **(B)** *NECTIN4* and *TROP2* (TACSTD2) expression levels grouped according to the Base47 molecular classification (Wilcoxon test). **(C)** Correlation of the *NECTIN4* and *TACSTD2* gene expression (Pearson test).

Supplementary Figure 3 FGFR3 expression in PDXs and sensitivity to anti-FGFR of FGFR3 mutated PDXs. **(A)** FGFR3 protein levels (Western blot) of the different PDX models. The urinary BCa cell line, RT112, was used as control for WT FGFR3 (black arrow) and FGFR3-TACC3 (white arrow) protein expression. Beta-actin was used as loading control. Asterisk indicates non-specific band. **(B)** Mice with established *FGFR3*-mutated (FGFR3-MT) PDXs (67-270 mm3) were treated with a pan-FGFR inhibitor (BGJ398). Control mice were treated with vehicle alone (n = 7 to 10 animals per group). Tumor size was measured at the indicated time points. Data are presented as mean ± SEM. Results were compared using Mann-Whitney test. **(C)** EGFR, ERBB2 and ERBB3 expression (RT-qPCR analysis) in L987 model at the end of treatment in mice treated with vehicle or with the pan-FGFR inhibitor BGJ398.

Supplementary Table 1 Clinical and histological characteristics of patient tumors and matched PDXs.

Supplementary Table 2 Short Tandem repeat (STR) profiling of patient tumors and matched PDX(s).

Supplementary Table 3 List of the sequenced genes in the next-generation targeted sequencing panel.

Supplementary Table 4 Next-generation sequencing results (mutations, copy number alterations, microsatellite status and tumor mutational burden).

## Abbreviations

PDXs, patient-derived xenografts; NMIBC, non-muscle-invasive bladder cancer; MIBC, muscle-invasive bladder cancer; FGFR, fibroblast growth factor receptor; EGFR, epidermal growth factor; SCC, squamous cell carcinoma; UC, urothelial cell carcinoma; UTUC, upper urinary tract urothelial carcinoma.
