# Supplementary figures and images for: Integrated molecular and pharmacological characterization of patient-derived xenografts from bladder and ureteral cancers identifies new potential therapies
Source: Front Oncol. 2022 Aug 11;12:930731. doi: 10.3389/fonc.2022.930731 (PMC9405192; doi:10.3389/fonc.2022.930731)

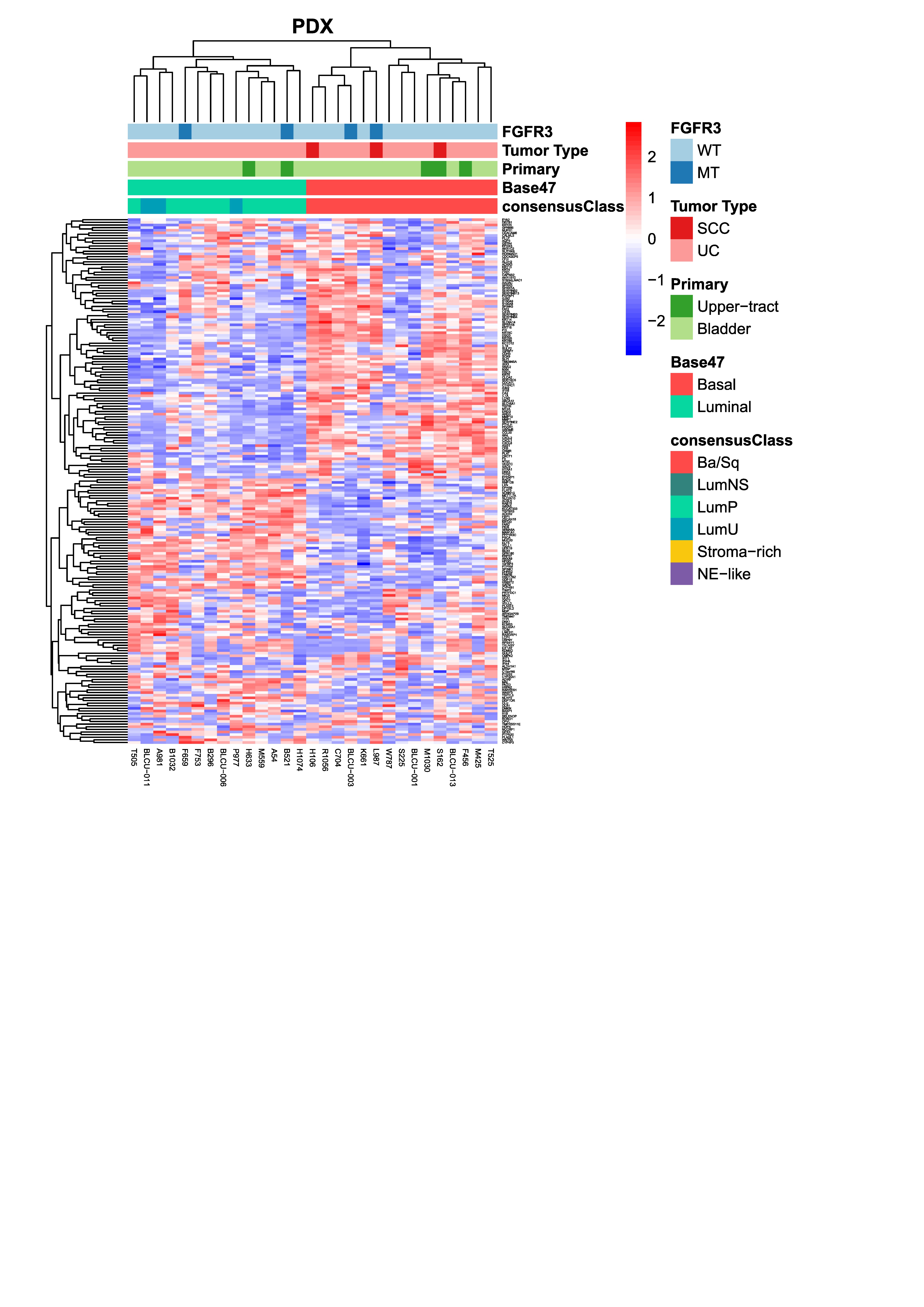

Supplement: Supplementary Figure 1 — Unsupervised hierarchical clustering and heatmap of PDX samples based on genes with the most variant expression (n=200). PDX identifiers indicated at the bottom. Classifications indicated according to legend on the right (as in Figure 2). [file Image_1.jpg]

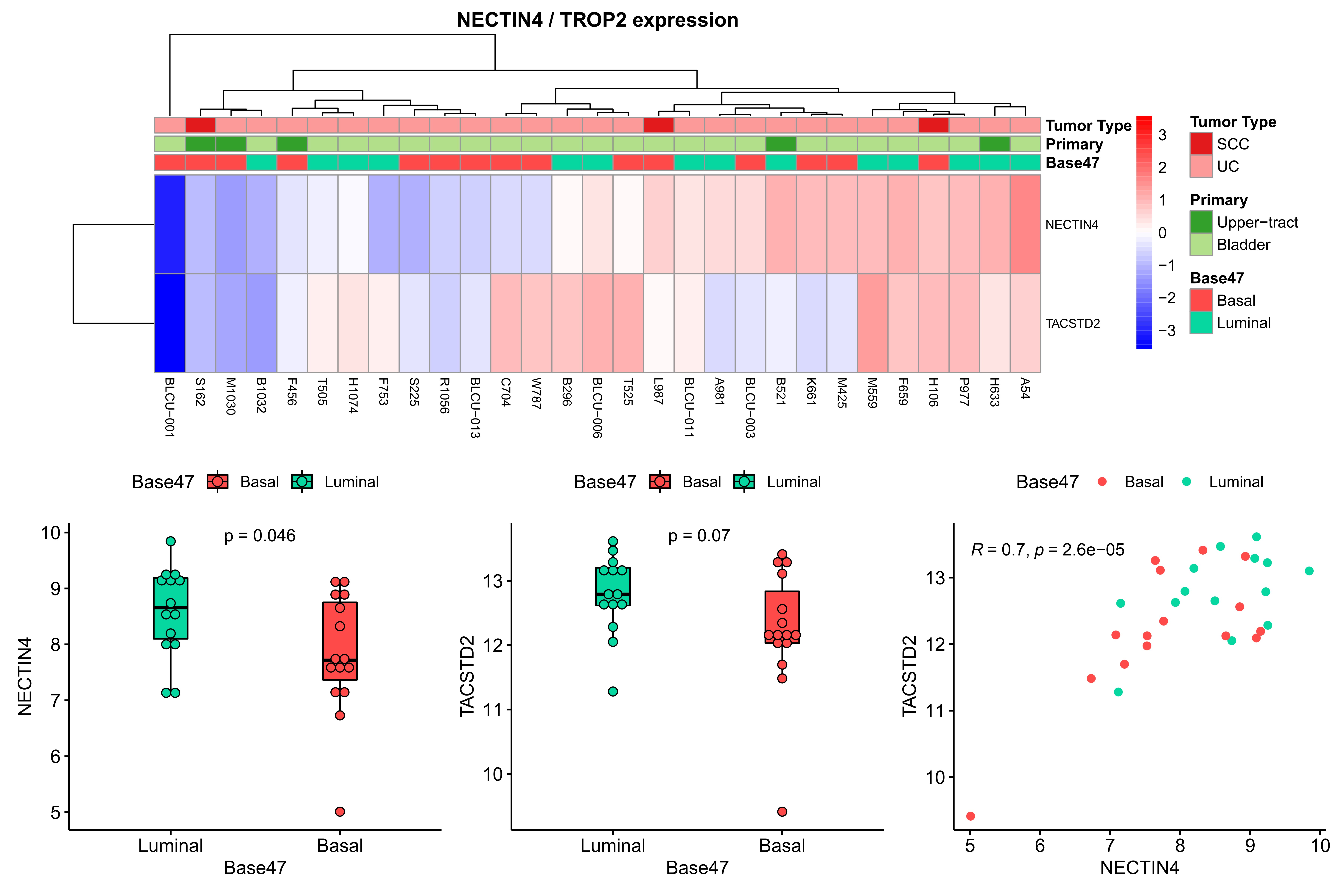

Supplement: Supplementary Figure 2 — Expression of NECTIN4 and TROP2 (TACSTD2) in PDXs. (A) Heatmap of PDX samples based on NECTIN4 and TACSTD2 gene expression. (B) NECTIN4 and TROP2 (TACSTD2) expression levels grouped according to the Base47 molecular classification (Wilcoxon test). (C) Correlation of the NECTIN4 and TACSTD2 gene expression (Pearson test). [file Image_2.jpg]

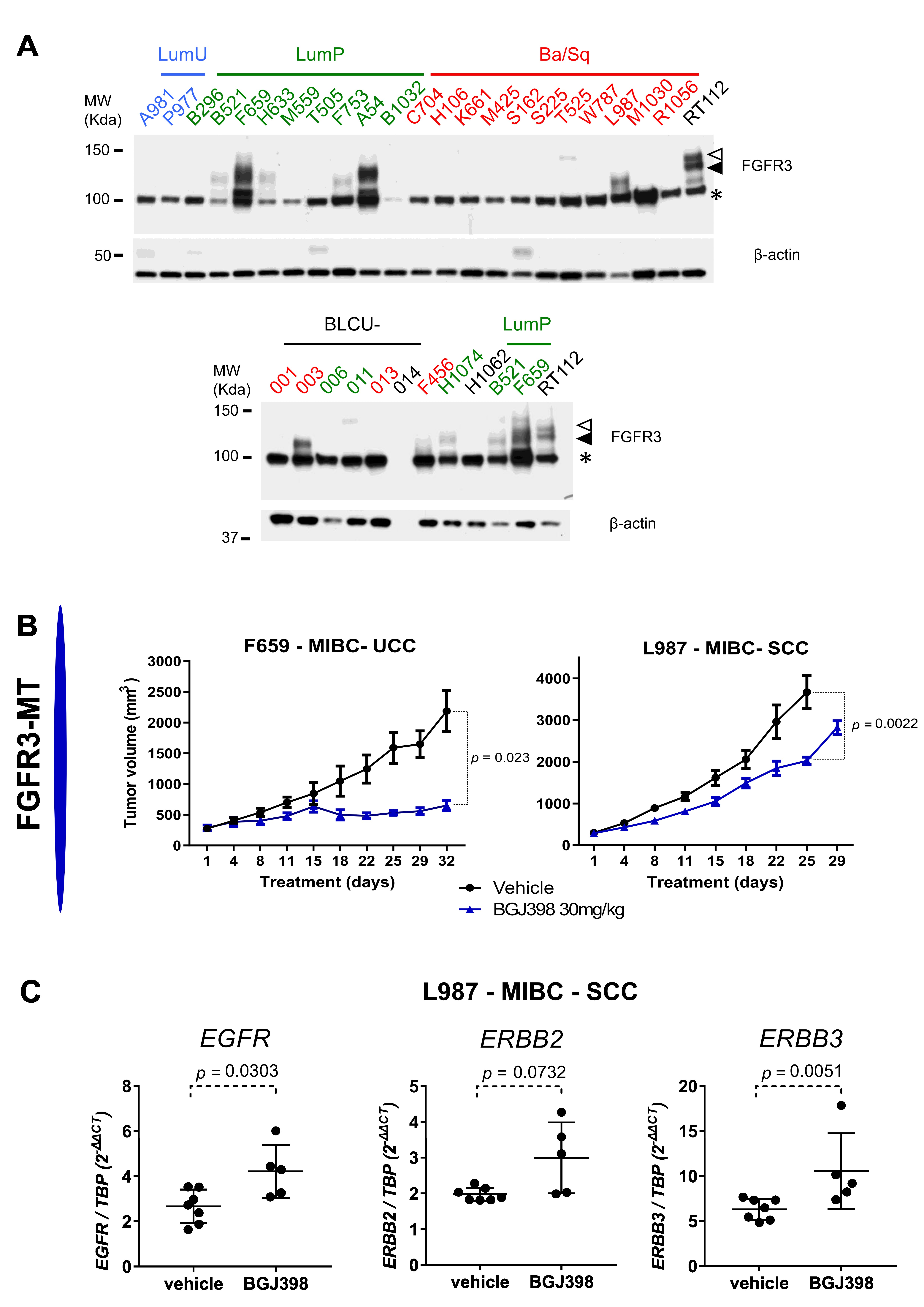

Supplement: Supplementary Figure 3 — FGFR3 expression in PDXs and sensitivity to anti-FGFR of FGFR3 mutated PDXs. (A) FGFR3 protein levels (Western blot) of the different PDX models. The urinary BCa cell line, RT112, was used as control for WT FGFR3 (black arrow) and FGFR3-TACC3 (white arrow) protein expression. Beta-actin was used as loading control. Asterisk indicates non-specific band. (B) Mice with established FGFR3-mutated (FGFR3-MT) PDXs (67-270 mm3) were treated with a pan-FGFR inhibitor (BGJ398). Control mice were treated with vehicle alone (n = 7 to 10 animals per group). Tumor size was measured at the indicated time points. Data are presented as mean ± SEM. Results were compared using Mann-Whitney test. (C) EGFR, ERBB2 and ERBB3 expression (RT-qPCR analysis) in L987 model at the end of treatment in mice treated with vehicle or with the pan-FGFR inhibitor BGJ398. [file Image_3.jpg]
